# Identification of miRNA-mRNA Modules in Colorectal Cancer Using Rough Hypercuboid Based Supervised Clustering

**DOI:** 10.1038/srep42809

**Published:** 2017-02-21

**Authors:** Sushmita Paul, Petra Lakatos, Arndt Hartmann, Regine Schneider-Stock, Julio Vera

**Affiliations:** 1 Department of Bioscience & Bioengineering, Indian Institute of Technology Jodhpur, India; 2 Experimental Tumorpathology, Institute of Pathology, University Hospital of Friedrich-Alexander-University Erlangen-Nürnberg, Germany; 3 Institute of Pathology, University Hospital of Friedrich-Alexander-University Erlangen-Nürnberg, Germany; 4 Laboratory of Systems Tumor Immunology, Department of Dermatology, Erlangen University Hospital and Friedrich-Alexander-Universität Erlangen-Nürnberg, Erlangen, Germany

## Abstract

Differences in the expression profiles of miRNAs and mRNAs have been reported in colorectal cancer. Nevertheless, information on important miRNA-mRNA regulatory modules in colorectal cancer is still lacking. In this regard, this study presents an application of the RH-SAC algorithm on miRNA and mRNA expression data for identification of potential miRNA-mRNA modules. First, a set of miRNA rules was generated using the RH-SAC algorithm. The mRNA targets of the selected miRNAs were identified using the miRTarBase database. Next, the expression values of target mRNAs were used to generate mRNA rules using the RH-SAC. Then all miRNA-mRNA rules have been integrated for generating networks. The RH-SAC algorithm unlike other existing methods selects a group of co-expressed miRNAs and mRNAs that are also differentially expressed. In total 17 miRNAs and 141 mRNAs were selected. The enrichment analysis of selected mRNAs revealed that our method selected mRNAs that are significantly associated with colorectal cancer. We identified novel miRNA/mRNA interactions in colorectal cancer. Through experiment, we could confirm that one of our discovered miRNAs, hsa-miR-93-5p, was significantly up-regulated in 75.8% CRC in comparison to their corresponding non-tumor samples. It could have the potential to examine colorectal cancer subtype specific unique miRNA/mRNA interactions.

Colorectal Cancer (CRC) has become one of the most serious malignancies worldwide. CRC is the third most frequently diagnosed cancer and the fourth leading cause of cancer related death worldwide, accounting for 1.2 million new cases and 6,08,000 deaths annually^1^. It is a highly heterogeneous disease and prognosis of patients with advanced CRC remains poor. Large number of studies have shown that miRNAs play an important role in the development and progression of CRC^2^,^3^,^4^,^5^.

MicroRNA (miRNAs) are a class of short approximately 22-nucleotide non-coding RNAs processed from hairpin precursors of ~70 nt (pre-miRNA), extracted, in turn, from primary transcripts (pri-miRNA) found in many plants and animals. Their functional roles have been studied in many crucial biological processes, including development, differentiation, apoptosis and cell proliferation^6^,^7^,^8^,^9^, as well as in numerous human diseases, such as chronic lymphocytic leukemia, fragile X syndrome, and various types of cancers^2^,^10^,^11^,^12^. The binding of miRNAs to the 3′ untranslated region of the mRNA leads to the down regulation of its mRNA expression. miRNAs along with lncRNAs have been found to be associated with complex diseases. In this regard, several computational models are developed^13^,^14^,^15^,^16^,^17^. Aberrant miRNA expression has been observed in CRC. The mechanism of miRNAs in the development and progression of the CRC is still not clear. Identification of mRNAs regulated by miRNAs might help to understand the biological roles of miRNAs^18^. However, simple sequence alignment approach may lead to false positive or insignificant miRNA-mRNA relation. In this regard, when considering the expression data of both, miRNAs and mRNAs, as biomarkers we could identify disease-associated miRNAs-mRNAs interactions/modules. Moreover, using both types of expression data could help to identify functional relationships between miRNAs and mRNAs and such modules will unravel key mechanisms involved in cancer regulation.

With this background, several studies have been conducted to identify potential miRNA-mRNA modules using expression data sets. Most of the methods used *t*-test or other univariate methods to identify a set of differentially expressed miRNAs and mRNAs. Mostly, Pearson correlation value has been used to create miRNA and mRNA networks. Szeto *et al*. developed a method to identify an mRNA-miRNA regulatory network in nasopharyngeal carcinoma model systems^19^. Student’s *t*-test has been employed for miRNA and mRNA expression data to identify a miRNA-mRNA regulatory network in CRC^20^. Zhou *et al*. used Significance Analysis of Microarrays and limma algorithms to select differentially expressed miRNAs and mRNAs which were further integrated to form a network for CRC^21^. A novel statistical method has been developed in ref. 22 to identify miRNA-mRNA interactions specific to certain cancer types. On the other hand, a causality discovery-based method has been used to uncover the regulatory relationship between miRNA and mRNA in an epithelial-to-mesenchymal transition data set^23^. Joung *et al*.^24^ used population-based probabilistic learning method to identify miRNA-mRNA modules. Connecting rule-based method has been employed on miRNA and mRNA expression data to identify miRNA-mRNA modules in an HCV data set^25^. However, all these methods selected differentially expressed miRNAs or mRNAs based on certain criteria and later the selected biomarkers were used to construct modules. These methods did not select groups of functionally similar miRNAs/mRNAs that could further classify clinical outcome and they did not consider the similarity/redundancy between the selected miRNAs/mRNAs. The method mentioned in ref. 25 selected groups of miRNAs/mRNAs that could differentiate negative samples from positive ones. The limitation of this method was that it did not consider the similarity between the selected sets of miRNAs/mRNAs in each rule/cluster. Also, the maximum size of each rule/cluster was 2 meaning that each cluster/rule contained only 2 miRNAs/mRNAs.

One of the main problems in expression data analysis is uncertainty. Some of the sources of this uncertainty include imprecision in computations and vagueness in class definition. With this background, the rough set^26^ provides a mathematical framework to capture uncertainties associated with human cognition process^27^,^28^. In refs 29, 30, 31, rough sets have been successfully used to identify differentially expressed genes from gene expression data. Importance of rough sets is also shown in clustering analysis. Rough sets were used to design clustering algorithms^32^,^33^ to identify groups of co-expressed genes from gene microarray data sets. They were also used to design methods to select differentially expressed miRNAs^31^,^34^ and to clustering functionally similar miRNAs^35^. A supervised clustering method^36^ based on rough sets has been also developed to group miRNAs whose average expression could further classify clinical outcome.

In this paper, we present a computational approach to identify miRNA-mRNA modules in CRC. It is a two step approach, at first miRNA rules/clusters were generated using the rough hypercuboid based supervised clustering algorithm^36^ (RH-SAC). Instead of selecting single miRNAs or mRNAs based on certain criteria as described by Fu *et al*.^20^ the RH-SAC algorithm generates clusters of functionally similar miRNAs/mRNAs whose coherent expression further classifies the samples efficiently. The RH-SAC algorithm overcomes all these above mentioned issues. In contrast to ref. 25, the RH-SAC generated *d* number of rules/clusters. Therefore, the classification accuracy of support vector machine was used to select best miRNA rules/clusters. To compute prediction accuracy of support vector machine both leave-one-out cross-validation (LOOCV) and 10-fold cross-validation (10-CV) were used. For each of these rules, mRNA targets of every miRNA were selected from experimentally validated miRTarBase database^37^. The expression data of the genes which were selected as miRNA targets were used for further analysis. Instead of selecting only negatively correlated miRNAs and mRNAs the proposed approach selected both types of positive and negative interactions since some studies have shown that both, negative and positive correlations, might exist between miRNAs and mRNAs^38^,^39^,^40^,^41^. Hence, RH-SAC is able to select potential miRNA-mRNA regulatory networks. The RH-SAC algorithm was also applied on reduced mRNA expression data to generate rules. In a second step, the obtained miRNA and mRNA rules were integrated to form a potential miRNA-mRNA regulatory module. Functional enrichment analyses, disease association, fisher’s test, and correlation analysis have been performed to relate the modules with important signaling pathways and processes of CRC. Survival analysis of the mRNAs of one of the modules was also done to evaluate the mRNAs in terms of clinical importance. The effectiveness of the proposed approach over other biomathematical method is also demonstrated in this paper especially in terms of enrichment analysis, statistical analysis, and survival analysis of obtained genes. For two miRNAs the expression patterns was compared in patient material of tumor and non-tumor tissues. The most important network motif involving hsa-miR-27a-3p and p53/CDKN1A genes has been analysed and discussed in detail.

## Materials and Methods

This section describes the data set that has been used in this study and the method to select rules/cluster of miRNAs/mRNAs. It also includes description of molecular studies.

### Data sets used

The miRNA and mRNA expression of CRC data sets were downloaded from the Gene Expression Omnibus (GEO) with accession number GSE35982. The miRNA expression data set that has also been used by Fu *et al*.^20^ contains 8 CRC tissues and their corresponding adjacent normal tissues. The CRC group consisted of micro-satellite stable, moderately differentiated, non-mucinous adenocarcinoma and their corresponding samples from adjacent normal tissues. In pre-processing step the Agilent Feature Extraction (FE) software version 9.5.3 was used for background subtraction and with-in array normalization. The between-array normalization and filtering on flags (IsGeneDetected, WellAboveNeg) was carried out by the RMA algorithm using the AgiMicroRNA^42^ R package. Finally, 126 miRNAs were selected for generation of rules.

The mRNA expression data was also generated from the same 8 paired samples as that for miRNA expression profiling. The mRNA expression data was processed using Agilent Feature Extraction software (version 9.5.3). The Feature extraction software generated raw expression data that was later imported into R using LIMMA package^43^. Within and between array normalization using quantile scaling algorithm was done using LIMMA package and the log 2 based data was then filtered on flags (IsFound, IsWellAboveBG, IsSaturated). A total 43,376 mRNAs were used in this study.

### Rough hypercuboid based supervised clustering

The rough hypercuboid based supervised clustering (RH-SAC)^36^ algorithm has been used to select potential rules/clusters of miRNAs/mRNAs. This algorithm discovers groups of biomarkers, which are not only functionally similar but their average expression values can efficiently discriminate samples. The unique rough hypercuboid based supervised similarity measure of this algorithm helps the algorithm to group functionally similar biomarkers.

Let 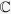 = 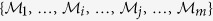 set of features and 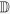 is the class label. Let 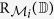 be the relevance of feature 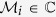 with respect to class label 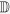. The relevance uses information about the class labels and is thus a criterion for supervised clustering. It is a metric that helps to judge the discriminatory capability of a feature^36^. It’s value ranges from 0 to 1. Near the value is to 1, better the discriminatory power of the feature is. The supervised clustering algorithm starts with a single feature 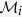 that has the highest relevance value with respect to class labels. An initial cluster 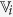 is then formed by selecting the set of features 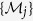 from the whole set 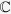. In the formation of initial cluster 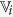 the similarity value 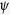 as described in ref. 36 is calculated between 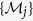 and the representative of cluster 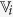. If the similarity value 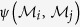 is greater than the pre-defined threshold *δ* then 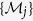 becomes part of 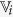, where


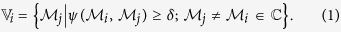


Hence, the cluster 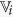 represents the set of features of 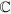 that have the supervised similarity values with the feature 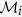 greater than a pre-defined threshold value *δ*. The cluster 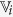 is the coarse cluster corresponding to the feature 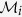, while the threshold *δ* is termed as the radius of cluster 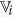.

Once the initial cluster 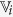 is formed, the cluster representative is refined by adding other features to the cluster. By searching among the features of cluster 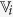, the current cluster representative is merged and averaged with one single feature such that the augmented cluster representative 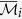 increases the relevance value. Here, two augmented cluster representatives are generated by averaging 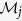 (using (2)) or its complement with the features of 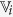 (using (3)). The merging process is repeated until the relevance value can no longer be improved. Instead of averaging all features of 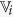, the augmented feature 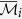 is computed by considering a subset of features 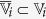 that increase the relevance value of cluster representative 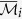. The set of features 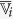 represents the finer cluster of the feature 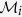. While the generation of coarse cluster reduces the redundancy among features of the set 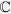, that of finer cluster increases the relevance with respect to class labels. After generating the augmented cluster representative 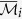 from the finer cluster 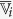, the process is repeated to find more clusters and augmented cluster representatives by discarding the set of features 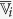 from the whole set 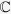.

The main steps of the supervised feature clustering algorithm are reported next.

- Let 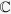 be the set of features of the original data set, while 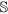 and 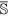 are the set of actual and augmented attributes, respectively, selected by the feature clustering algorithm.
- Let 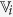 be the coarse cluster associated with the feature 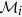 and 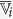, the finer cluster of 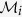, represents the set of features of 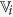 those are merged and averaged with the feature 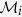 to generate the augmented cluster representative 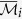.

1. Initialize 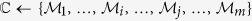, 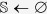, and 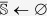.
2. Calculate the rough hypercuboid based relevance value 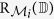 of each feature 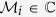 as mentioned in ref. 36.
3. Repeat the following nine steps (steps 4 to 12) until 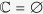 or the desired number of attributes are selected.
4. Select feature 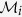 from 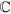 as the representative of cluster 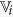 that has highest rough hypercuboid based relevance value^36^. In effect, 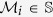, 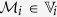, 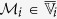, and 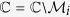.
5. Generate coarse cluster 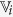 from the set of existing features of 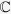 satisfying the following condition^36^:
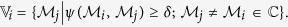
6. Initialize 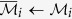.
7. Repeat following four steps (steps 8 to 11) for each feature 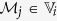.
8. Compute two augmented cluster representatives by averaging 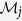 and its complement with the features of 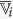 as follows:
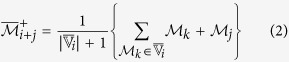

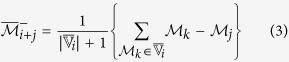
9. The augmented cluster representative 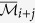 after averaging 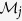 or its complement with 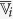 is as follows:
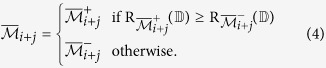
10. The augmented cluster representative 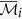 of cluster 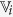 is 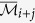 if 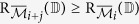, otherwise 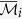 remains unchanged.
11. Select attribute 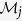 or its complement as a member of the finer cluster 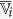 of attribute 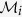 if 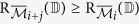.
12. In effect, 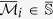 and 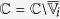.
13. Stop.

### Support Vector Machine

In the current study, the support vector machine (SVM)^44^ is used to select potential miRNA/mRNA rules generated by the RH-SAC algorithm. The SVM is a margin classifier that draws an optimal hyperplane in the feature vector space; this defines a boundary that maximizes the margin between data samples in different classes, therefore leading to good generalization properties. A key factor in the SVM is to use kernels to construct nonlinear decision boundary. In the present work, linear kernels is used. The source code of the SVM has been downloaded from Library for Support Vector Machines (www.csie.ntu.edu.tw/~cjlin/libsvm/).

### Identification of miRNA-mRNA modules

**Figure 1:**
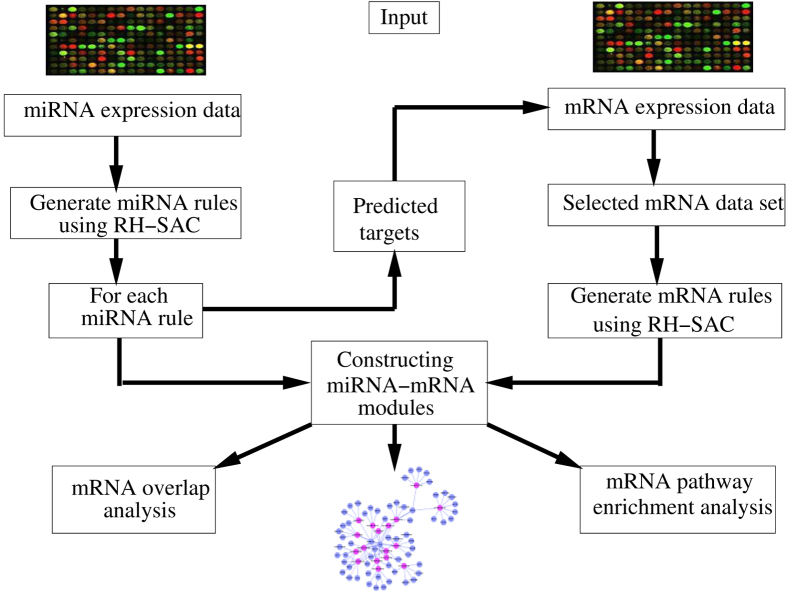
Schematic flow diagram of the proposed approach for identification of miRNA-mRNA modules.

The first step towards generating miRNA-mRNA modules was to select miRNA clusters/rules whose average expression can 100% accurately classify the samples using SVM classifier. For generation of miRNA rule/cluster the RH-SAC algorithm was used as this algorithm generates a cluster of functionally similar miRNAs whose average expression can further classify samples. Hence, the method selects groups of miRNAs that are functionally similar as well as highly differentially expressed. To obtain the classification accuracy of the SVM both leave-one-out cross-validation (LOOCV) and 10-fold cross-validation (10-fold CV) were used. The experimentally validated miRNA target database, miRTarBase^37^ was further used to select targets for each miRNA of a cluster/rule with 100% LOOCV accuracy. Subsequently, mRNA data sets for each miRNA cluster/rule were designed by considering only those mRNAs that were also a target according to the miRTarBase. On these reduced mRNA expression data set for each miRNA cluster/rule, the RH-SAC algorithm was employed in order to select groups of mRNAs that are functionally similar as well as differentially expressed. It leads to generation of mRNA clusters/rules. The mRNA clusters/rules that achieved 100% LOOCV accuracy were selected for further analysis. Finally, the mRNA rules and their miRNA rules were combined to form a miRNA-mRNA regulatory module. For visualizing the generated miRNA-mRNA regulatory modules an open source software platform termed as Cytoscape^45^ has been used. The proposed in-silico approach is illustrated in Fig. 1.

### Molecular studies

#### Patient material

Formalin-fixed in paraffin embedded tissue samples of 29 patients with colorectal tumors were provided by the Institute of Pathology, University Hospital Erlangen. Use of patient material was approved by the ethical committee of the University Hospital Erlangen. All experiments were performed in accordance with relevant guidelines and regulations. The tumor group consisted of 16 males and 13 female patients; the average age was 68.7 years (range 46–88 years); 4 patients has TNM stage I, 11 had TNM stage II, 12 had TNM stage III, and 2 had TNM stage IV; 15 patients with and 14 patients were without lymph node metastases, respectively.

#### RNA/miRNAextraction

To extract RNA/miRNA from formalin-fixed in paraffin embedded tissues the RecoverAll™ Total Nucleic Acid Isolation Kit (Ambion) was used according to instructions of the manufacturer.

#### Reverse transcription

Reverse transcription was performed using the miScript II RT kit (Qiagen, Hilden, Germany) according to instructions of the manufacturer.

#### Quantitiative Real-time PCR

Real-time PCR was performed using the miScript SYBR Green PCR kit (Qiagen, Hilden, Germany) according to instructions of the manufacturer. The 25 *μ*l PCR volume contained 10 ng cDNA in each reaction and the miRNAs specific primers (hsa-miR-93-5p MIMAT0000093: 5′CAAAGUGCUGUUCGUGCAGGUAG; hsa-miR-223-3p MIMAT0000280: 5′UGUCAGUUUGUCAAAUACCCCA), miScript Universal Primer with QuantiTect SYBR Green PCR Master Mix.The results were detected on a CFX96 Real-time PCR Detection system (Biorad, Hercules, California). The reactions were incubated at 95 °C for 15 min, followed by 40 cycles of 94 °C for 15 s, 55 °C for 30 s and 70 °C for 30 s. For normalization of the raw data the human RNU6_2 miScript Primer set was used.

### Colorectal cancer patient samples for survival analysis

Gene signatures of module 23 were evaluated for understanding their clinical importance. In this regard, survival analysis^46^ was performed for the genes of module 23. For conducting survival analysis we used SurvExpress^47^ tool. To evaluate the biomarkers in several conditions, the dataset was chosen to reflect patients suitable for the test. Hence, we selected relevant colorectal cancer patient samples and corresponding clinical information of Colon Rectal Adenocarcinoma TCGA data available at the SurvExpress tool. We used Kaplan-Meier method to asses the survival. Survival analyses were performed using Cox regression model. We performed Cox hazard regression analysis based on gene expression profiles and stratified patients based on death information.

## Experimental Results and Discussions

In this section the performance of the proposed approach to identify miRNA-mRNA regulatory modules is presented. The maximum number of clusters selected by the RH-SAC algorithm was 50. The *δ* parameter of the RH-SAC algorithm^36^ controls the size of a cluster. In this study, the *δ* parameter value was set to 0.9 as the best results (in terms of classification accuracy of SVM) obtained at *δ* = 0.9. Each data set was pre-processed by standardizing each sample to zero mean and unit variance. In the following sections importance of all miRNA rules, mRNA rules, miRNA-mRNA modules is discussed.

### Discriminatory rules from the miRNA expression data

**Figure 2:**
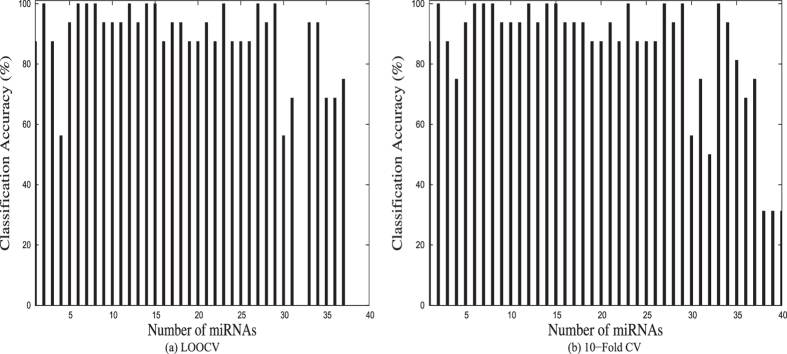
Classification accuracies of obtained miRNA clusters.

**Figure 3:**
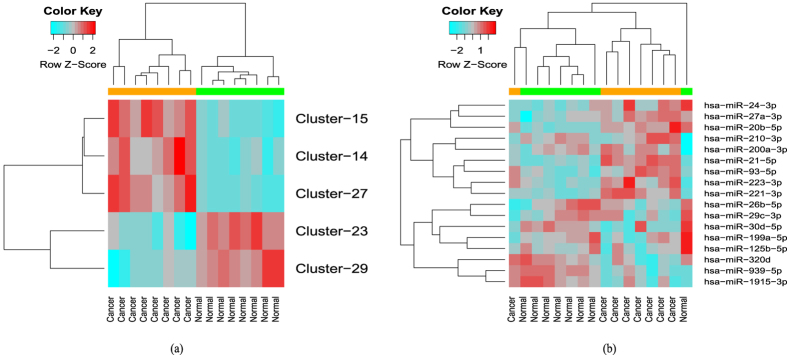
Heatmap of 5 miRNA clusters/rules and selected 17 miRNAs: (**a**) similar expression patterns were observed in cancer samples compared to normal samples based on the average expression values of 5 miRNA rules. Green colored band represents normal cases whereas orange colored band represents cancer. (**b**) 5 miRNA rules contain in total 17 differentially expressed miRNAs, based on the expression values of these miRNAs, samples are very well classified into cancer and normal except two cases.

The RH-SAC algorithm generated 5 clusters/rules that achieved 100% LOOCV classification accuracy and each cluster contained more than one miRNA in the cluster. On the other hand, 6 clusters/rules that achieved 100% 10-fold CV classification accuracy were generated. Figure 2 represents the LOOCV and 10-fold CV classification accuracies of all miRNA clusters. From the figure it can be seen that clusters 14, 15, 23, 27, and 29 reached 100% LOOCV classification accuracy. Whereas, clusters 14, 15, 23, 27, 29, and 33 reached 100% 10-fold CV classification accuracy. Moreover, these clusters contained more than one miRNA. Clusters that achieved 100% accuracy and contained only one miRNA were excluded from further study. From the figure it is seen that 10-fold CV generated more number of clusters having 100% classification accuracy. Five out of six clusters that achieved 100% 10-fold CV were also detected by LOOCV approach. The target genes of the Cluster-33 of 10-fold CV were not found to be significantly enriched (results not shown). In this study results of LOOCV were used for further analysis. Total 17 miRNAs belonging to one of the 5 clusters having 100% LOOCV accuracy were selected. Figure 3 represents the heatmaps of the average expression values of 5 miRNA clusters/rules and all the 17 miRNAs belonging to one of the rules/clusters. From both heatmaps of miRNA data it is clear that the obtained rules and miRNAs effectively discriminate between cancer samples and normal samples. Only one normal sample and one cancer sample in the heatmap of 17 miRNAs were not clustered accurately.

Table 1 represents the selected miRNAs and their corresponding rules. From the table it is seen that rules 1 and 2 contain 2 miRNAs each, while rules 3, 4, and 5 contain 4, 5, and 4 miRNAs, respectively. The number of miRTarBase targets of each miRNA is also mentioned here. The expression values of selected miRTarBase targets were further used to generate reduced mRNA data sets for each miRNA rule. From Table 1 it is seen that the final number of mRNAs is smaller than targets identified by miRTarBase. The same miRNA data set has been also used by Fu *et al*.^20^. They used student’s *t*-test for selection of differentially expressed miRNAs followed by Pearson’s correlation for miRNA and mRNA expression profiles using a *p*-value less than 0.05 to be statistically significant. Their method selected 22 dysregulated miRNAs in the eight CRC samples. We found an overlap of 4 miRNAs with their list of dysregulated miRNAs. In addition, our approach selected a group of functionally similar miRNAs that were also differentially expressed.

### Discriminatory rules from the mRNA expression data

**Figure 4:**
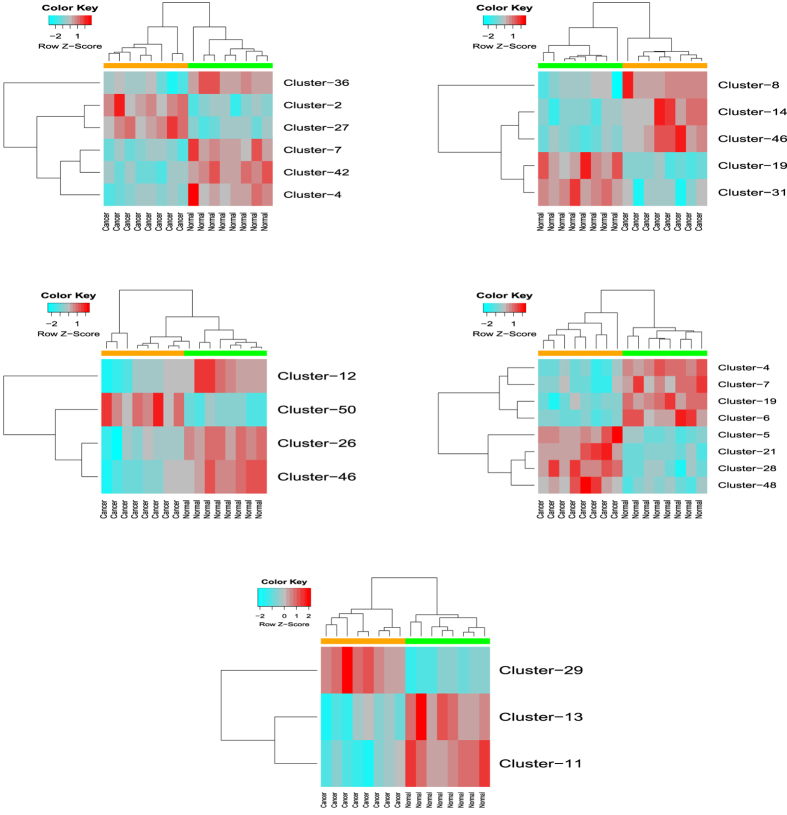
Heatmaps of selected mRNA clusters: 5 heatmaps correspond to 5 miRNA rules, normal samples (green colored band) and cancer samples (orange colored band) are perfectly classified.

The original mRNA expression data set was reduced into smaller data sets for each miRNA rule as per the information of miRTarBase. Some of the target mRNAs from miRTarBase were not present in the expression data set so they were excluded from further analysis. On each mRNA data set that corresponds to one of the miRNA rule the RH-SAC algorithm was applied. Finally, those mRNA clusters were selected that achieved 100% LOOCV classification accuracy. Also, the clusters contained more than one mRNA. The number of mRNA clusters generated from each reduced mRNA expression data set that corresponds to one of the five miRNA rules is presented in Table 2. Total number of mRNAs in each reduced mRNA expression data set is also given in Table 2 and total number of mRNAs for each mRNA rule is mentioned in Table 2. Figure 4 represents heatmaps of 5 mRNA rules. From this figure it is evident that the RH-SAC algorithm selected a group of mRNAs/genes whose average expression could efficiently classify samples.

### miRNA-mRNA regulatory modules

**Figure 5:**
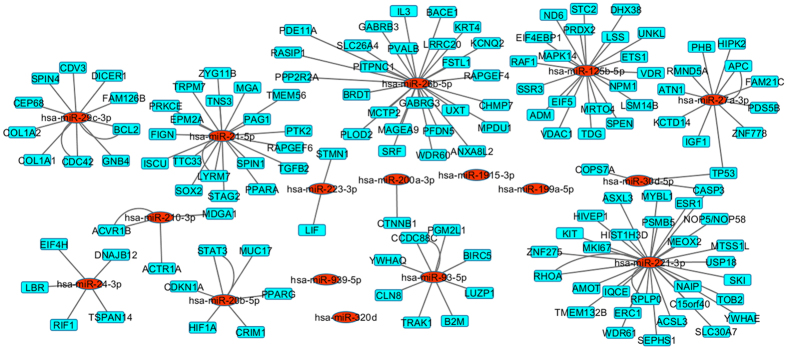
miRNA-mRNA regulatory interaction networks: interactions between miRNA and mRNA of each module are shown in this figure. Here, red colored nodes are miRNAs whereas blue colored nodes are mRNAs. For 4 miRNAs (red nodes) no targets were selected by the RH-SAC algorithm.

In total 5 miRNA rules (containing 17 miRNAs) were selected and the significant mRNA rules (containing 141 mRNA) were merged to form 5 miRNA-mRNA regulatory modules. Cytoscape software^45^ was used to generate the interactions between miRNAs and mRNAs. While the red coloured nodes denote miRNAs, blue coloured nodes represent mRNAs. Figure 5 represents all obtained miRNA-mRNA regulatory interaction networks. Many miRNAs and mRNAs in this regulatory module were related to CRC. A detailed explanation will be given in the subsequent section.

### Functional enrichment analysis of each miRNA-mRNA module

This section presents the functional enrichment analysis of each miRNA-mRNA module. For this analysis Functional annotation tool of DAVID^48^ has been used for both GO enrichment and KEGG pathway analysis. The enrichment *P*-value was corrected to control family-wide false discovery rate under certain rate (e.g., <0.05) with Benjamin multiple testing correction method. However, not all the modules could generate enriched modules at the given threshold. Hence, results for those modules were also reported here after relaxing the threshold. In this analysis rule 3 has been observed as the most significant rule.

**Figure 6:**
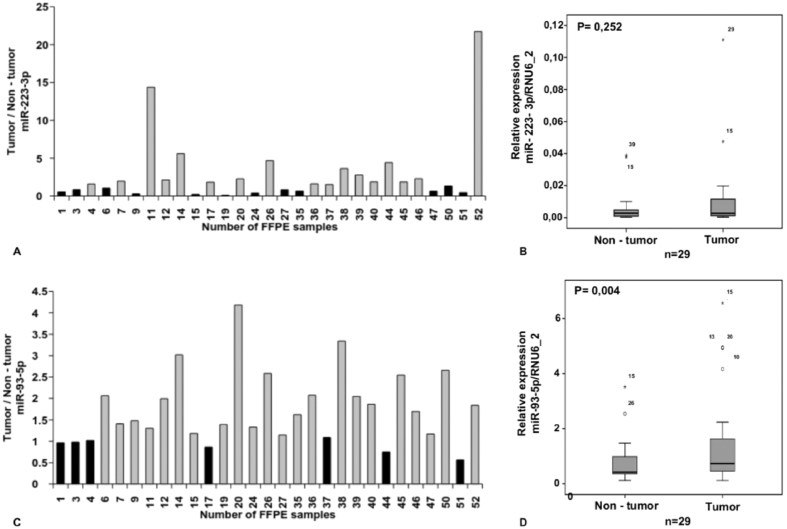
hsa-miR-223-3p was shown to be not significantly up-regulated in CRC when compared with their normal counterparts (**A**,**B**). hsa-miR-93-5p is significantly up-regulated in 75.8% CRC in comparison to their corresponding non-tumor samples (**C**,**D**).

**Figure 7:**
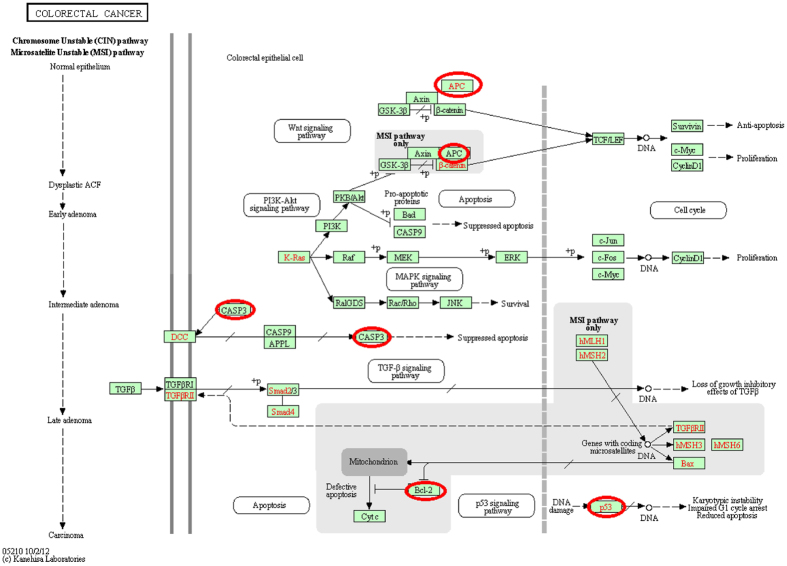
The colorectal cancer pathway^59^.

- In the module of hsa-miR-221-3p and hsa-miR-223-3p, none of the target mRNA/gene was found to be associated with any KEGG pathway significantly. However, after relaxing the threshold few pathways were obtained that were associated with the mRNAs/genes of the first module. The pathways are *Pathways in cancer, Neurotrophin signaling pathway, Cytokine-cytokine receptor interaction*, and *MAPK signaling pathway* and their uncorrected *P*-values were 0.18, 0.26, 0.47, and 0.48, respectively. On the other hand, several significant GO terms were obtained for this module and they were mainly related to cell cycle. hsa-miR-223-3p was shown to be up-regulated in CRC when compared with their normal counterparts (Table 3).
- We studied the hsa-miR-223-3p expression in 29 tumor/non-tumor pairs and found an up-regulation only in 17 of 29 (58%) CRC cases when compared to their non-tumor tissues. Overall, there was no significant difference (Fig. 6A,B) may be due to the small sample cohort. hsa-miR-223-3p expression did not correlate with any clinicopathological factors such as sex, age, grade, TNM or the presence of lymph node metastases.
- In the module of hsa-miR-93-5p and hsa-miR-21-5p, the genes were associated with *cell cycle* KEGG pathway with uncorrected *P*-value < 0.05 and *pathways in cancer* with uncorrected *P*-value of 0.08. On the other hand, the genes of this module were associated with the GO terms that have uncorrected *P*-value ≤ 0.05. The GO terms were *eye development, neuron differentiation, regulation of cell development, protein targeting, negative regulation of cell differentiation*, and *sensory organ development*. Although most studies showed an up-regulation of hsa-miR-93-5p in CRC, there are no consistent reports about a tumor suppressor or oncogenic function of hsa-miR-93-5p (Table 4). Here we showed that hsa-miR-93-5p was significantly up-regulated in 29 CRC in comparison to the corresponding non-tumor samples (Fig. 6C,D). Furthermore, hsa-miR-93-5p expression was higher in advanced tumors (*P*-value = 0.053). Observing the same results, Yang *et al*.^49^ unravel a hsa-miR-93-5p mediated cell cycle repression. We could not show any correlation of hsa-miR-93-5p expression and immunohistochemical expression of the p21 cell cycle inhibitor.
- The third module containing hsa-miR-29c-3p, hsa-miR-20b-5p, hsa-miR-27a-3p, and hsa-miR-30d-5p was the most significant module, as this module contained many genes/mRNAs that were significantly associated with important GO terms and KEGG terms at a corrected *P*-value < 0.05. Figure 7 represents the *colorectal cancer* pathway and the circled genes are present in the obtained third module. The image was obtained by KEGG^50^ with permission. Other important significant KEGG pathways obtained were *pathways in cancer, p*53 *signaling pathway, Focal adhesion*, and *Prostate cancer*. Various cell cycle and other important GO terms were found to be associated with the genes of this module at a significant level. One interesting finding for this module using David software was that two of the genes of this module namely, *APC* and *TP*53 were found to be significantly associated with the disease ontology term *coloractal cancer* with uncorrected *P*-value of 0.0029.
- The genes/mRNAs of the fourth module were associated with *Leukacyte transendothelial migration, Tight junction, Neuroactive ligand-receptor interaction* with uncorrected *P*-value of 0.19, 0.21, and 0.37, respectively. However, some GO terms were found to be significantly enriched for this module. The significant GO terms were *cell-cell signaling, protein folding, synaptic transmission*, and *transmission of nerve impulse*. These GO terms had uncorrected *P*-value ≤ 0.05.
- The last module contained genes/mRNAs that were associated with the KEGG terms, namely *MAPK signaling pathway* and *Acute myeloid leukemia* with uncorrected *P*-value of 0.0774 and 0.0981, respectively. The genes/mRNAs with uncorrected *P*-value < 0.05 were also found to be associated with some GO terms like *positive regulation of erythrocyte differentiation, regulation of erythrocyte differetiation* etc.

These results indicate that the proposed approach selected important miRNA-mRNA regulatory modules in CRC. In comparison to Fu *et al*.^20^ who found in total 58 genes and among them few were exclusively connected to only *Wnt signaling pathway*, our approach identified different important regulatory modules whose genes were significantly associated with colorectal cancer pathway and many other important KEGG pathways.

### Overlap with disease related genes

The genes of the most significant module 3 were further analysed in terms of degree of overlapping with a known cancer related gene list^51^. This list contains 742 cancer related genes, which were collected from the Cancer Gene Census of the Sanger Centre, Atlas of Genetics and Cytogenetic in Oncology^52^, and Human Protein Reference Database^53^ from a total number of 18,491 genes in Illumina Ref-8 whole-genome expression Bead Chip. Table 5 represents the statistical significance test of the genes of our module 3 with respect to the cancer related gene list. In total 10 genes (*APC, ATN*1, *BCL*2, *CDKN*1*A, COL*1*A*1, *COL*1*A*2, *DICER*1, *PHB, PPARG*, and *TP*53) out of 29 genes of module 3 were found to be overlapped with the known cancer related genes. Considering the results of Fu *et al*.^20^ only 3 out of 58 target mRNAs (*KIAA*1549, *LPP*, and *MAP*2*K*4) overlapped with the cancer gene list. Using the Fisher’s exact test the *P*-value of genes obtained by Fu *et al*. method was 0.5057, while our proposed approach generated a *P*-value of 1.021*e*–07. The RH-SAC algorithm selected functionally similar genes that were also differentially expressed. Since, genes of module 3 of the RH-SAC method were more similar to each other we were able to generate a lower *P*-value as compared to the Fu *et al*. method. Hence, it indicates that our approach has the potential to identify valuable miRNA-mRNA regulatory modules. In general we have to take into account that the used GEO data set was comprised of a specific histological subset of CRC but according to ref. 54 there is a gene expression-based subclassification of CRC. The authors suggest that the identified subclassification signatures could explain different and heterogeneous drug response in patient cohorts. Thus we might speculate that different molecular subtypes have also a different but characteristic miRNA signatures.

### Prognostic value of modules in colorectal cancer

Genes of Rule-23 were analysed to study their correlation with patient’s survival. Total 29 genes were studied. We investigated whether the combined elevated expression (as that in ref. 46) of 29 selected genes in colorectal adenocarcinoma patient samples, extracted from TCGA, was related to the prognosis of patients with colorectal cancer. Here, we compared the clinical importance of our obtained 29 gene signatures with the 19 gene signatures of colorectal cancer mentioned in ref. 55.

**Figure 8:**
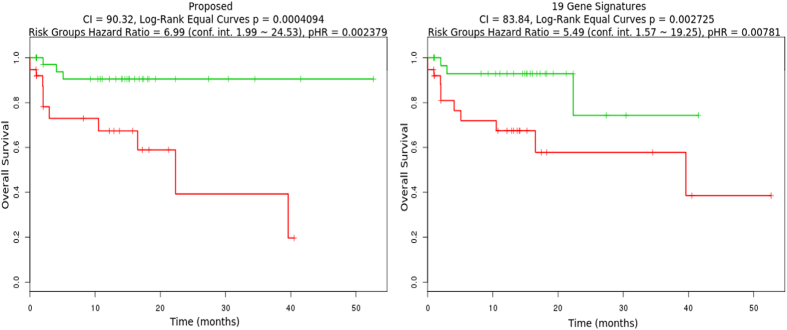
Comparison of Kaplan-Meier curves for patients with colorectal cancer plotted for combined expression of 29 genes obtained by proposed method and 19 genes mentioned in ref. 55.

For combined selected genes, we analysed the Kaplan-Meier survival curve as a first step in assessing the prognostic value of the corresponding genes in colorectal cancer. Patient samples were divided into two categories based on the combined expression of 29 genes. Patients (n = 75) having high expression values for the combined genes were grouped into high-expression group (red coloured curve) and remaining are grouped into low-expression group (green coloured curve) (n = 76). From the Kaplan-Meier survival analysis (Fig. 8) it is seen that the biomarkers obtained from proposed method as well as 19 biomarkers mentioned in ref. 55 are significantly able to separate two risk groups characterized by differences in their gene expression. From the figure it is seen that higher expression of the genes leads to poor overall survival of the patients. However, the log-rank test, concordance index (CI), hazard ratio (HR), P-value of hazard ratio (pHR), number of genes having significant beta coefficient from the cox fitting are better in the proposed method compared to the biomarkers mentioned in ref. 55. From the Fig. 8, it is seen that the proposed method performed better in terms of log-rank test, concordance index (CI), hazard ratio (HR), P-value of hazard ratio (pHR). In addition to that four genes of proposed method were found to be having significant beta coefficient from the cox fitting whereas only 2 genes were found to be significant for the method proposed in ref. 55. We also analysed 58 genes mentioned in ref. 20 for studying their clinical importance. Although the log-rank test value (4.600276e-06) and CI (1) value are better compared to the proposed method, the HR (1008588382), pHR (0.99) values obtained are abrupt and insignificant. Moreover, no genes were found having significant beta coefficient from the cox fitting. Therefore, the results indicate that the selected genes of module-23 are highly correlated with patient survival.

### Pearson correlation of miRNAs and mRNAs present in most significant module

It is very well known that miRNAs suppress the expression of mRNAs. However, recent studies show that this phenomenon is not always correct. In this section the positive and negative correlation of the miRNAs and mRNAs of Rule-3 is discussed since the members of this module were highly associated with colorectal cancer. Table 6 represents the Pearson correlation values of 4 miRNAs and 29 mRNAs. From Table 6 it is seen that most of the miRNA-mRNA pairs have a negative correlation indicating that these mRNAs are negatively regulated by their corresponding miRNAs. On the other hand, there are some miRNA-mRNA pairs having a positive correlation value. 18 cases have negative correlation coefficient values while, 13 cases have positive correlation coefficient. It suggests that some of miRNAs can positively regulate the mRNA by binding at the 5′ UTR of the mRNA^25^. hsa-miR-30d-5p has been already described as potential tumor suppressor^56^. In ref. 57 it has been shown that hsa-miR-30d-5p acts as a tumor suppressor and positively regulates *CASP*3. Therefore, the predicted positively regulated miRNA-mRNA interactions can be used to study so far unknown pathways in colorectal cancer.

### Network Analysis of Module 3

**Figure 9:**
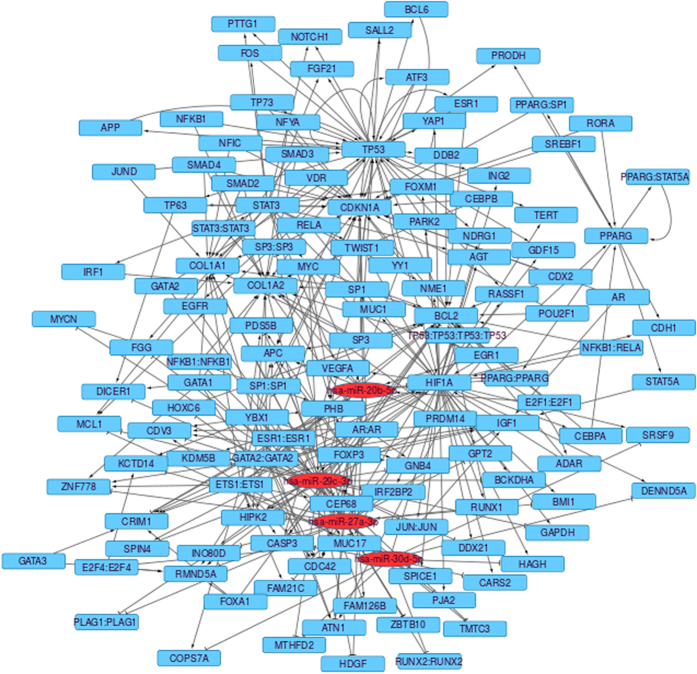
Extended network of miRNAs and mRNAs of module 3, containing 133 nodes and 349 edges. Red colored nodes are miRNAs whereas blue colored nodes are genes.

This section presents the network analysis of the obtained 4 miRNAs and 29 mRNAs of the most significant module 3 selected by the RH-SAC algorithm. This initial module/network was further extended by querying the databases miRTarBase^37^, HTRIdb^58^, TRANSFAC^59^, and TransmiR^60^. Following are the main steps of the network extension:

- Interactions between 33 seeds or the 4 miRNAs or 29 mRNAs were identified using the above mentioned databases. This forms the primary network.
- The primary network is expanded with one layer using information of the before mentioned databases. Now, the network contains 1560 nodes and 11,128 edges.
- Remove all non-seed nodes of degree zero and one. The size of this network is 1518 nodes and 11,086 edges.
- Remove all non-seed miRNAs. The size of this network is 1393 nodes and 7073 edges.
- Next, remove all nodes from the network that are not directly connected to the input miRNAs and mRNAs. Hence, the size of the network reflects 1393 nodes and 1540 edges.
- Finally, remove all non-seed nodes of degree zero and one. The size of the final network reflects 133 nodes and 349 edges. Figure 9 represents the extended network.

The final version of the network was further analyzed to identify regulatory motifs like feedback (FBLs) and feedforward loops (FFLs), with the help of the Cytoscape plugin NetDS^61^. The transcription factors and miRNAs are responsible for the combinatorial regulation of gene expression at transcription level and post-transcription level, respectively^62^. The network shown in Fig. 9 is a complex inter-regulatory network. To understand the mechanism of this complex network it is highly important to understand and analyze its key role players, which recur throughout the network^63^. The recurring elements of the complex network are known as network motifs^64^. A network motif generally contains three or more interacting components that are able to perform elementary signal processing functions. A network motif can be depicted as the smallest functional modules of the network and, by logically connecting them, the total complexity of the original network can be recovered^62^. Network motifs can be categorised into feedback loops (FBLs) and feedforward loops (FFLs)^65^. Among them one may identify positive and negative FBLs and coherent and incoherent FFLs. Negative feedback loops, including those containing miRNAs, can induce homeostasis and fast signal termination, while positive feedback loops can induce self-sustained signal amplification and all-or-nothing activation^65^. A specially interesting case of positive FBLs for miRNAs circuits are those connecting a miRNA with its own repressor transcription factor. Under some circumstances, they can behave as toggle switches. Coherent feedforward loops, those in which a target gene is consistently regulated by a TF directly and via TF controlled miRNA, have two effects. On the one hand, they can transiently delay the response of the target gene to the double regulation exerted by the TF. Also, they can reinforce the repression of the target gene. Incoherent miRNA-mediated FFLs can work as gene noise buffers, a feature that suggests miRNAs as important players in the fine-tuning of genetic circuits. All these types of motifs represent different ways of regulation of gene expression. In this work, we identified several coherent FFLs using NetDS and they may be studied further to understand the pathogenesis of colorectal cancer. MiRNA-mediated gene regulatory networks are widely studied to understand the key roles in the pathological processes of cancer^66^,^67^. Zhao *et al*.^68^ identified synergetic regulatory networks mediated by oncogenic miRNAs and TFs in serous ovarian cancer. Sun *et al*.^69^ studied various FFLs and demonstrated oncogenic potential of those FFLs in glioblastoma. FFLs are also studied in T-cell acute lymphoblastic leukaemia^70^, osteosarcoma^71^, and henceforth.

**Figure 10:**
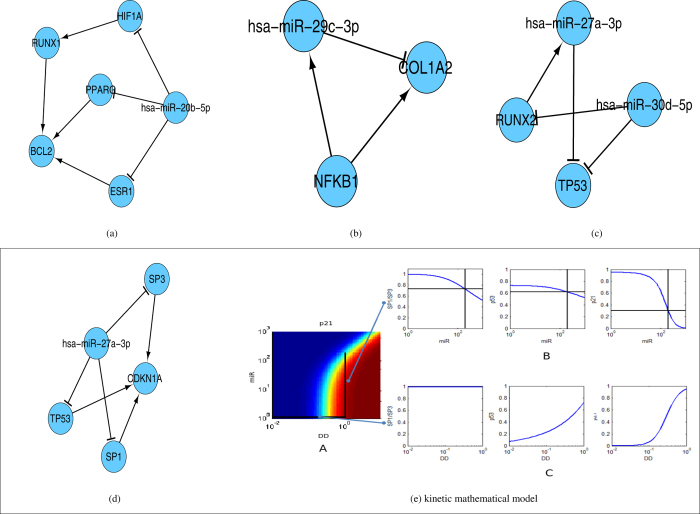
Coupled feedforward loops (**a**–**d**) along with kinetic mathematical model (**e**) accounting for the hsa-miR-27a-3p (**d**) regulation of *p*21 during DNA damage.

In the current study, total 4 coherent feedback loops and 178 coherent feedforward loops were obtained. However, no feedback loops with a specific miRNA were obtained. But, a number of feedforward loops with miRNAs were selected using NetDS. A few of them are presented in Fig. 10. The obtained regulatory motifs can be further converted into a detailed mechanistic model. One of the FFLs (Fig. 10(d)) is described next. The subnetwork obtained is a triple coherent feedforward loop, in which hsa-miR-27a-3p represses in parallel three transcription factors collectively promoting the expression of *p*21. Upon DNA damage (DD) *p*53 triggers the expression of *CDKN*1*A* (a.k.a *p*21), a well described cell cycle regulator that stops the proliferation of damage cells until the DNA gets repaired and therefore is primarily considered to be tumor suppressor. The *p*53 mediated expression of *p*21 requires *SP*1 and *SP*3^72^,^73^. Is it known that in physiological conditions miRNAs promote a rather mild repression of their targets. However, we hypothesize that the combination of miRNA regulation with non linear motifs like feedforward loops can modify this behavior. To investigate this hypothesis, we derived a kinetic mathematical model accounting for the hsa-miR-27a-3p regulation of *p*21 during DD. Description of the mathematical model is provided in Supplementary material (S1). To this end we modified and integrated two previous models on the dynamics of *p*53^74^ and *p*21^75^ during DD in cancer cells. The model is provided as Supplementary Material. In the model, hsa-miR-27a-3p can promote a mild downregulation of its targets (20% reduction by approximately 100 fold increase in expression). In Fig. 10(e) we systematically modified the values of the model input variables accounting for miRNA (miR) expression and DNA damage and computed the expression values of *SP*3/1, *p*53 and *p*21. When DD is increased under basal miRNA levels, *p*53 and subsequently *p*21 expression are triggered, while *SP*1/3 expression remains unchanged Fig. 10(c,e). Under moderate DD values, an increase in the expression of hsa-miR-27a-3p provokes a mild repression of its targets *SP*1, *SP*3 and *p*53 Fig. 10(b,e). However, through the combination of the coherent feedforward loop and the synergism in the regulation of *p*21, the predicted impact on the expression of *p*21 gets amplified achieving up to a 70% downregulation of *p*21 for a 200 fold increase in hsa-miR-27a-3p Fig. 10(b,e). Taken together, our computational analysis suggests that the existence of the coherent feedforward loop repression of *p*21 by hsa-miR-27a-3p can selectively amplify its repression compared to other hsa-miR-27a-3p targets.

**Figure 11:**
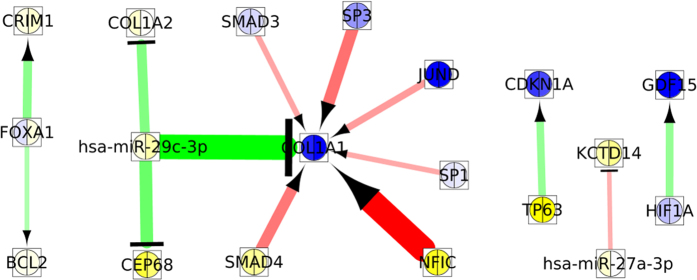
Condensed network resulting from the extended network highlighting links across which the largest changes are observed.

Next, ExprEssence^76^ is used to condense extended network so that they contain only those links between miRNAs/mRNAs, which have a large amount of expression changes. These links are called most differentially altered. The obtained results may help in building hypotheses about the startup or the shutdown of interactions, stimulations and inhibitions. Figure 11 represents various condensed networks obtained from the extended network. The red colour link represents positive (startup) and green colour link represents negative (shutdown) side of the spectrum of link score values. The higher expression value of mRNA/miRNA is represented by blue colour and lower expression value is represented by yellow colour. The edges keeping the 3% quantiles of the most strongly differentially altered interactions are taken as condensed networks. The startup of the stimulation of *COL*1*A*1 by *NFIC* and shutdown of the inhibition of *COL*1*A*1 by hsa-miR-29c-3p are the largest changes. The downregulation of the inhibition of *COL*1*A*1 and *COL*1*A*2 may trigger the higher expression of *COL*1*A*1 and *COL*1*A*2 in cancer compared to normal counterparts, since there is a lower expression of hsa-miR-29c-3p in cancer state than in normal state. Similar phenomena have been seen in nasopharyngeal carcinomas^77^. From Fig. 11, it is seen that the startup of stimulations is in the center of the protein *COL*1*A*1, whose expression is increased in cancer state compared to the normal state. Cooperative regulation of *COL*1*A*1 by *NFIC* and *SP*1 has been observed by ref. 78. In ref. 78, it was described that the higher expression of *SP*1 inhibits the expression level of *COL*1*A*1. When the expression of *SP*1 decreases from 10.70 to 10.61 the expression of *COL*1*A*1 increases from 13.94 to 15.19. On the other hand, since *NFIC* increases its promoter activity: when the expression of *NFIC* increases from 7.19 to 7.57 the expression of *COL*1*A*1 also increases from 13.94 to 15.19. The up-regulation of the *SP*3-mediated *COL*1*A*1 stimulation has been also demonstrated by Saitta *et al*.^79^ in human dermal fibroblasts. *SMAD*3 and *SMAD*4 mediated stimulation of *COL*1*A*1 is described in ref. 80. The increased inhibition of *KCTD*14 by hsa-miR-27a-3p is reflected by lower expression of *KCTD*14. The expression of both genes *HIF*1*A* and *GDF*15, decreases, indicating the shut down of stimulation.

## Conclusion

The main contribution of this paper lies in identification of potential miRNA-mRNA regulatory modules in colorectal cancer using corresponding miRNA and mRNA expression data. The important rules/clusters of each type of biomarkers were identified using the rough hypercuboid based supervised attribute clustering algorithm. These clusters contained co-expressed miRNAs/mRNAs whose average expression could effectively classify the samples. The biological importance of the obtained modules has been discussed in detail. The mRNAs selected by our approach were found to be significantly associated with many important KEGG pathways and GO terms especially colon cancer. The survival analysis of mRNAs of one of the modules established their clinical importance. We identified novel miRNA/mRNA interactions in CRC and suggest to use this approach in larger sample groups. We believe that our method has the potential to examine subtype specific unique miRNA/mRNA interactions. Indeed, the genes/mRNAs of the most significant module 3 were significantly overlapping with already known cancer gene lists. Interestingly, both, positive and negative types of relationships between miRNAs and mRNAs have been observed in correlation based analysis.

## Additional Information

**How to cite this article:** Paul, S. *et al*. Identification of miRNA-mRNA Modules in Colorectal Cancer Using Rough Hypercuboid Based Supervised Clustering. *Sci. Rep.* **7**, 42809; doi: 10.1038/srep42809 (2017).

**Publisher's note:** Springer Nature remains neutral with regard to jurisdictional claims in published maps and institutional affiliations.

## Supplementary Material

Supplementary Information

## Figures and Tables

**Table 1:** Description of Each miRNA Rule.

Rule/Cluster	Selected miRNA	No. of Target mRNAs from miRTarBase	No. of mRNAs in Each Cluster Finally Used
Rule-1	hsa-miR-221-3p	250	249
	hsa-miR-223-3p	20	
Rule-2	hsa-miR-93-5p	611	823
	hsa-miR-21-5p	491	
Rule-3	hsa-miR-29c-3p	105	245
	hsa-miR-20b-5p	17	
	hsa-miR-27a-3p	103	
	hsa-miR-30d-5p	61	
Rule-4	hsa-miR-200a-3p	37	1787
	hsa-miR-199a-5p	20	
	hsa-miR-1915-3p	7	
	hsa-miR-24-3p	315	
	hsa-miR-26b-5p	1638	
Rule-5	hsa-miR-125b-5p	300	323
	hsa-miR-320d	2	
	hsa-miR-939-5p	2	
	hsa-miR-210-3p	54	

**Table 2:** The Target mRNAs and Their Rules for Each miRNA Rule.

No. of mRNAs in Each miRNA Cluster Finally Used	mRNAs Selected by RH-SAC	Total Number of mRNA Cluster
249	33	6
823	29	5
245	33	4
1787	32	8
323	24	3

**Table 3:** hsa-miR-223-3p Expression in Various Studies.

hsa-miR-223-3p	Cancer type/Profile	References
1	CRC and plasma UP	81
2	CRC UP	82
3	CRC UP	83
4	CRC and plasma UP	84
5	CRC UP	20
6	Ovarian UP	85
7	Bladder cancer UP	86
8	Gastric cancer UP	87
9	Cholangiocarcinoma UP	88
10	Hepatocellular carcinoma DOWN	89

**Table 4:** hsa-miR-93-5p Expression in Various Studies.

miR-93-5p	Cancer type/Profile	References
1	Colon UP	90
2	Colorectal UP	91
3	Colorectal carcinoma DOWN	92
4	Colorectal UP	49
5	CRC plasma UP	93
6	CRC UP	94
7	Hepatocellular UP	95
8	Non-small lung UP	96
9	Lung UP	97
10	Laryngeal squamous cell carcinoma UP	98
11	HNSCC UP	99
12	Ovarian carcinoma DOWN	100

**Table 5:** Overlap with cancer gene list.

		Yes	No	Total
Fu *et al*.	yes	3	55	58
	no	739	17694	18433
Proposed	yes	10	19	29
	no	732	17730	18462
Total		742	17749	18491

**Table 6:** Pearson correlation coefficient values between miRNAs and mRNAs of module-3.

	hsa-miR-29c-3p	hsa-miR-20b-5p	hsa-miR-27a-3p	hsa-miR-30d-5p
APC	—	—	−0.1593	—
ATN1	—	—	0.0434	—
BCL2	−0.3821	—	—	—
CASP3	—	—	—	0.2322
CDC42	−0.0699	—	—	—
CDKN1A	—	0.0252	—	—
CDV3	0.3099	—	—	—
CEP68	−0.6030	—	—	—
COL1A1	−0.2175	—	—	—
COL1A2	−0.2585	—	—	—
COPS7A	—	—	—	−0.0419
CRIM1	—	−0.1046	—	—
DICER1	0.0437	—	—	—
FAM126B	−0.0549	—	—	—
FAM21C	—	—	−0.1660	—
GNB4	−0.5777	—	—	—
HIF1A	—	−0.2779	—	—
HIPK2	—	—	−0.2292	—
IGF1	—	—	−0.5196	—
KCTD14	—	—	0.0652	—
MUC17	—	0.0073	—	—
PDS5B	—	—	0.2830	—
PHB	—	—	0.4625	—
PPARG	—	0.3125	—	—
RMND5A	—	—	−0.2091	—
SPIN4	0.3670	—	—	—
STAT3	—	−0.3452	—	—
TP53	—	—	0.1937	−0.0408
ZNF778	—	—	0.2113	—
