# Supplementary material for: Identification of miRNA-mRNA Modules in Colorectal Cancer Using Rough Hypercuboid Based Supervised Clustering
Source: Sci Rep. 2017 Feb 21;7:42809. doi: 10.1038/srep42809 (PMC5318911; doi:10.1038/srep42809)
Supplement: Supplementary Information [file srep42809-s1.pdf]

Identification of miRNA-mRNA Modules in Colorectal Cancer Using Rough Hypercuboid Based Supervised Clustering

Sushmita Paul, Petra Lakatos, Arndt Hartmann, Regine Schneider-Stock, and Julio Vera

SREP-16-40454

```
% Mathematical model for the triple coherent feedforward,loop, in which hsa-miR-27a-3p represses
in parallel SP1, SP3 and p53,
% three transcription factors collectively promoting the expression of CDKN1A (a.k.a. p21)
% Model contained in "Identification of miRNA-mRNA Modules in Colorectal Cancer Using
% Rough Hypercuboid Based Supervised Clustering" (Paul et al. 2016)
%
%
% Model parameters
   g1=0.25;              % assumed miR repression follows a power-law rate equation (PMID:
17399948)...
                         % with mild single miRNA target represssion, such as target has basal
expression...
                         % (Tgt=1) at miRNA level miR=1, and 80% of basal (Tgt=.8) at miR=100
   X= 80;                % assumed like in g1
   g2= 4.9;              % assumed p21 mRNA synthesis follows a hill-equation in the product
SP1*SP3*p53,...
                         % such as p21 mRNA synthesis gets 10% of maximum for SP1*SP3*p53=0.25...
                         % and 99% for SP1*SP3*p53=1
   km= 0.39;             % assumed like in g2
   kdsp1= -log(0.5)/6;   % reference PMID: 19245816
   kdsp3= -log(0.5)/4;   % PMID: 17685427
   kdp53= 1.7;           % PMID: 22010756
   kdm2m= 0.9;           % PMID: 22010756
   kdm2= 0.8;            % PMID: 22010756
   kdp21m= 0.12;         % PMID: 22798498
   kdp21= 1.39           % PMID: 22798498
%
%
% Input variables (representative values)
   miR=1;     % Expression level for miR-27a-3p
   DD=1;      % DNA damage
%
%
% Time-dependent variables
   SP1=y(1);  % SP1
   SP3=y(2);  % SP3
   p53=y(3);  % p53
   M2m=y(4);  % MDM2 mRNA
   M2=y(5);   % MDM2
   p21m=y(6); % p21 mRNA
   p21=y(7);  % p21
%
%
% Model equations
   dy(1)= kdsp1*(1 + miR/X).^-g1 - kdsp1*SP1;         % SP1' (normalized)
   dy(2)= kdsp3*(1 + miR/X).^-g1 - kdsp3*SP3;         % SP3' (normalized)
   dy(3)= kdp53*DD*(1 + miR/X).^-g1 - kdp53*M2*p53;   % p53' (normalized)
   dy(4)= kdm2m*p53 - kdm2m*M2m;                      % M2m' (normalized)
   dy(5)= kdm2*M2m - kdm2*M2;                         % M2'  (normalized)
   dy(6)= kdp21m*(SP1*SP3*p53).^g2./(km.^g2...
      + (SP1*SP3*p53).^g2) - kdp21m*p21m;             % p21m'(normalized)
   dy(7)= kdp21*p21m - kdp21*p21;                     % p21' (normalized)
```
